# A seven-gene prognosis model to predict biochemical recurrence for prostate cancer based on the TCGA database

**DOI:** 10.3389/fsurg.2022.923473

**Published:** 2022-09-05

**Authors:** Yijun He, Jinxiong Zhang, Zhihao Chen, Kening Sun, Xin Wu, Jianhong Wu, Lu Sheng

**Affiliations:** Department of Urology, Huadong Hospital Affiliated to Fudan University, Shanghai, China

**Keywords:** prostate cancer, biochemical recurrence, prognosis model, the cancer genome atlas (TCGA), nomogram

## Abstract

**Background:**

The incidence rate of prostate cancer is increasing rapidly. This study aims to explore the gene-associated mechanism of prostate cancer biochemical recurrence (BCR) after radical prostatectomy and to construct a biochemical recurrence of prostate cancer prognostic model.

**Methods:**

The DEseq2 R package was used for the differential expression of mRNA. The ClusterProfiler R package was used to analyze the functional enrichment of Gene Ontology (GO) and Kyoto Encyclopedia of Genes and Genomes (KEGG) to explore related mechanisms. The Survival, Survminer, and My.stepwise R packages were used to construct the prognostic model to predict the biochemical recurrence-free probability. The RMS R package was used to draw the nomogram. For evaluating the prognostic model, the timeROC R package was used to draw the time-dependent ROC curve (receiver operating characteristic curve).

**Result:**

To investigate the association between mRNA and prostate cancer, we performed differential expression analysis on the TCGA (The Cancer Genome Atlas) database. Seven protein-coding genes (*VWA5B2*, *ARC*, *SOX11*, *MGAM*, *FOXN4*, *PRAME*, and *MMP26*) were picked as independent prognostic genes by regression analysis. Based on their Cox coefficient, a risk score formula was proposed. According to the risk scores, patients were divided into high- and low-risk groups based on the median score. Kaplan–Meier plot curves showed that the low-risk group had a better biochemical recurrence-free probability compared to the high-risk group. The 1-year, 3-year, and 5-year AUCs (areas under the ROC curve) of the model were 77%, 81%, and 86%, respectively. In addition, we built a nomogram based on the result of multivariate Cox regression analysis. Furthermore, we select the GSE46602 dataset as our external validation. The 1-year, 3-year, and 5-year AUCs of BCR-free probability were 83%, 82%, and 80%, respectively. Finally, the levels of seven genes showed a difference between PRAD tissues and adjacent non-tumorous tissues.

**Conclusions:**

This study shows that establishing a biochemical recurrence prediction prognostic model comprising seven protein-coding genes is an effective and precise method for predicting the progression of prostate cancer.

## Introduction

Prostate cancer is one of the most common cancers in the world. It is the fifth leading cause of cancer death among men in 2020. The incidence rate of prostate cancer is increasing rapidly with the development of the economy, the prolongation of life expectancy, and lifestyle changes (1). For clinically localized prostate cancer, radical prostatectomy (RP) and external beam radiation therapy (EBRT) are the most prevalent treatment strategies (2). Even though RP can have excellent control of the development of localized prostate cancer for most men, about 35% of patients will experience a detectable serum PSA elevation (3). BCR was defined as the patient who did not receive endocrine therapy and radiotherapy after RP and the prostate-specific antigen (PSA) ≥ 0.2 μg/L for two consecutive follow-ups. It does not indicate that patients have clinically recurrence or disease death (4). Nevertheless, BCR is related to the advancement of prostate cancer, distant metastases, and the overall mortality of the malignancy. Shelan et al. reported that 20%–40% of patients develop BCR after RP for localized prostate cancer (5). Blute et al. reported that after primary surgery, only around 30% of patients with BCR suffered a clinical recurrence (6). Although BCR is not a significant clinical outcome, it is still an effective endpoint that evaluates the curative effect after RP (7). Patients may not get into clinical recurrence even though they have BCR. However, most of the time, BCR is the biomarker of systematic recurrence, and the patients who have BCR have a higher risk of metastasis and mortality (6). In the clinical trial, an accurate predictive model for prostate cancer recurrence after RP is important in choosing the best treatment. A deep understanding of BCR, which plays a role in the development of prostate cancer, is crucial (7). Currently, there are fewer studies evaluating the influence of the protein-coding gene prognostic model on the biochemical recurrence of prostate cancer. Classical risk markers, such as PSA, pathology grade, and clinical stage, are still utilized to predict BCR (8). Briers et al. proposed that short PSA-DT, high final Gleason score following RP, short interval to biochemical failure (IBF) after radical radiotherapy (RT), and high biopsy Gleason score are the most dangerous factors to overall survival (4).

In this work, we aim to establish a novel prostate cancer prognostic model. The TCGA-PRAD dataset was accessed to retrieve mRNA sequencing data and relevant clinical information. Using Lasso and multivariate Cox regression analyses, seven protein-coding genes were identified as significant BCR indicators of prostate cancer. Eventually, we built a seven protein-coding gene model to predict the BCR of prostate cancer in patients.

## Materials and method

### Data processing

We downloaded the RNA-seq data from TCGA-PRAD, including 52 normal and 499 tumor tissues. The clinical information on TCGA-PRAD was also obtained. We download the RNA-seq dataset GSE46602 from the GEO database, including 14 normal and 36 tumor tissues. The clinical information of GSE46602 was also obtained. The patients with biochemical recurrence time less than 0.1 months were excluded. The table of clinical information on TCGA-PRAD is given in Table 1. The DEseq2 R package (9) was used for differential mRNA expression analysis. Differentially expressed mRNAs were chosen under the following criteria: |log fold change(FC)| > 2 and adjusted *P* value < 0.05. We analyzed the expression matrix and clinical information comprehensively and filtered the information once again. Last, we preserve 418 patients' tumor expression data and clinical information for prognosis analysis.

**Table 1:** Clinical characteristics of patients with PRAD from the TCGA database.

Variables	TCGA set, *n* = 418
Age	
<60	137
≥60	222
Missing	7
Overall survival	
Death	4
Alive	414
Prognosis	
Biochemical recurrence	52
Not biochemical recurrence	366

### Functional enrichment analyses

We used differential expression genes to make functional enrichment analyses. At the same time, we focused on the function of 51 protein-coding genes associated with biochemical recurrence. The ClusterProfiler (10) R package facilitates the functional enrichment analysis of GO and KEGG encoding genes. The GOplot (11) R package was used to visualize the result. The result of the functional enrichment analysis is presented in Supplement Tables 1, 2.

### Biochemical recurrence prognostic model construction

Log-rank analysis was conducted by using the Survival and Survminer R package, and 104 genes associated with BCR were identified with setting *P* < 0.05.Univariate Cox analysis was conducted using the Survival and Survminer R package, and 111 genes associated with BCR were identified with setting *P* < 0.05. Then, we ran an intersection, and 72 genes were found. There were 51 protein-coding genes among the 72 genes. We used these genes for further analysis. Next, Lasso Cox regression was employed to eliminate substantially overfitted protein-coding genes. Consequently, 20 protein-coding genes were screened using Lasso analysis. Finally, seven protein-coding proteins were selected by multivariate Cox regression analysis by the My.stepwise (12) R package (Supplement Table 3), and a risk score formula was derived based on the Cox coefficient. The formula is as follows:

$$\begin{array}{rl} \mathrm{Risk}\;\mathrm{Score}=\sum_{i=1}^{n}\beta i\times Ei\;\\ & (\beta i\;\mathrm{is}\;\mathrm{the}\;\mathrm{expression}\;\mathrm{of}\;\mathrm{the}\;\mathrm{gene}\;\\ \;\mathrm{in}\;\mathrm{the}\;\mathrm{model}\;\mathrm{and}\;Ei\;\mathrm{is}\;\mathrm{the}\;\mathrm{Coef}\;\mathrm{of}\;\mathrm{the}\;\mathrm{gene}) \end{array}$$

Then, according to the formula and based on the median score, all patients were separated into high- and low-risk groups. The Survminer R package was used to display the Kaplan–Meier plot curve.

### Construction of the timeROC curve and risk-model biochemical recurrence nomogram

The timeROC curve was generated by the timeROC (13) R package to analyze the 1-year, 3-year, and 5-year specificity and sensitivity of the prognostic risk model. Independent prognostic protein-coding genes of PRAD were included in the risk-model biochemical recurrence nomogram.

### Seven protein-coding expression levels between paired prostate cancer and adjacent nontumorous tissues

Eight paired prostate cancer and adjacent nontumorous tissue samples were obtained from the Huadong Hospital of Fudan University. The ethics committee of the Huadong Hospital of Fudan University has approved the use of clinical samples. The expression levels of mRNA were measured by quantitative real-time PCR. The primers of seven genes are listed in Supplement Table 4. The total RNA content of tissue was extracted by EZBioscience Tissue RNA Purification Kit PLUS. Next, mRNA was reverse transcribed into cDNA using TAKARA PrimeScript RT Master Mix (Perfect Real Time). Real-time PCR was performed by using Yeasen SYBR Green Master Mix (High Rox Plus) on an Applied Biosystems StepOnePlus Real-Time PCR System. The Ct value of each well was recorded, and relative quantification of the product was performed by using the 2^−ΔΔCt^ method. Differences between paratumor and PRAD groups were compared using Student's *t*-test.

## Results

### Identification of mRNA in TCGA-PRAD

**Figure 1:**
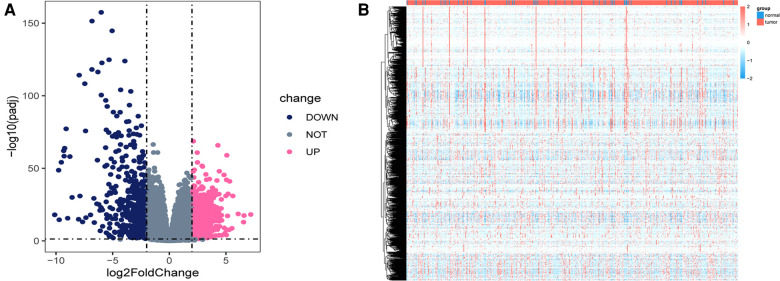
Differentially expressed genes in TCGA_PRAD. (**A**) Volcano plot showing the differentially expressed genes in TCGA-PRAD. The blue color indicates the downregulated genes, and the pink color indicates the upregulated genes. (**B**) Heatmap showing the differentially expressed genes between prostate cancer tissues and normal controls.

Using the DEseq2 R package, we found the mRNA between prostate cancer and adjacent normal tissue from TCGA-PRAD, with the adjusted *P* < 0.05 and |log fold change| > 2 as the threshold limit. There were 853 differential expression mRNAs in TCGA-PRAD. The distribution of mRNA was depicted using a volcano plot (Figure 1A). In addition, the heatmap displayed the expression profiles of mRNA in TCGA-PRAD (Figure 1B).

### GO and KEGG enrichment analysis for differential genes

**Figure 2:**
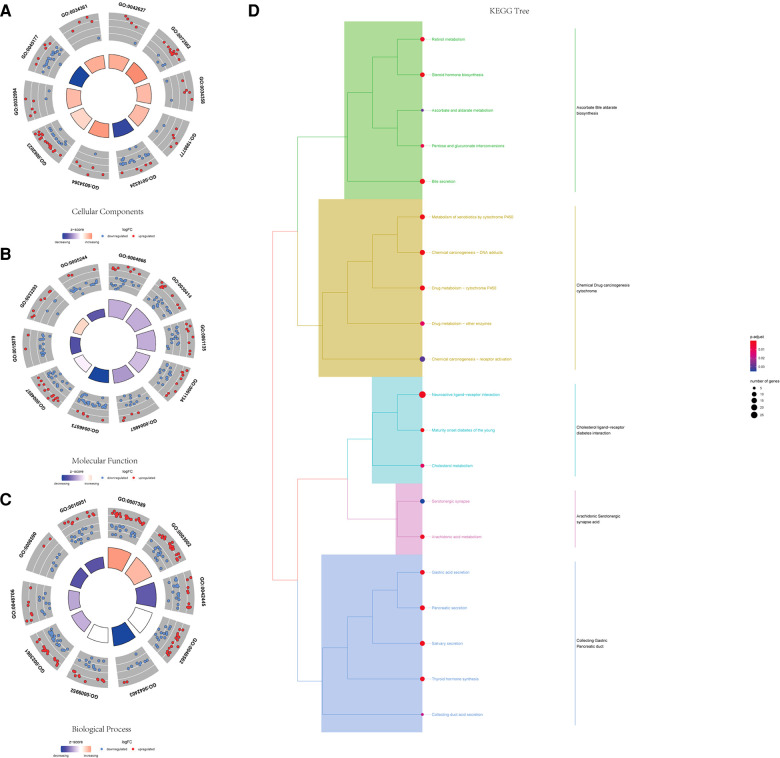
GO and KEGG functional enrichment of differential genes. (**A**) Top 10 significantly enriched biological process terms. (**B**) Top 10 significantly enriched cellular component terms. (**C**) Top 10 significantly enriched molecular function terms. (**D**) KEGG tree pathway analysis of the differential genes.

Using the ClusterProfiler R package, we evaluated the enrichment functions of these differentially expressed genes. We illustrated the top 10 terms under the condition of *P *< 0.05. As shown in Figure 2A, the cellular component (CC) of target genes was significantly enriched in chylomicron, blood microparticles, plasma lipoprotein particles, lipoprotein particles, and apical plasma membrane. The molecular function (MF) of the differential genes is associated with endopeptidase inhibitor activity, peptidase inhibitor activity, endopeptidase regulator activity, and peptidase regulator activity (Figure 2B). Moreover, it was discovered that the biological process (BP) contains the terms of the pattern specification process, regionalization, hormone metabolic process, and embryonic organ morphogenesis. These biological pathways may be associated with the progression of prostate cancer (Figure 2C). The KEGG results showed that differential genes were found to be involved in neuroactive ligand–receptor interaction, bile secretion, steroid hormone biosynthesis, drug metabolism-cytochrome P450, and metabolism of xenobiotics by cytochrome P450 (Figure 2D).

### Identification and selection of significant protein-coding genes

**Figure 3:**
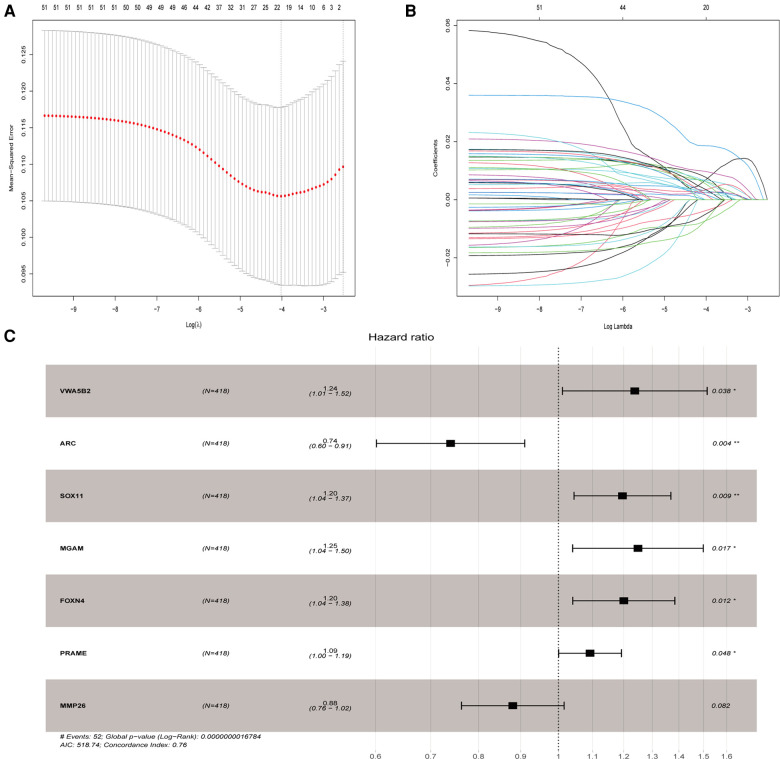
Lasso and univariate Cox analyses to identify independent prognostic and biochemical recurrence models. (**A**) Lasso regression to verify independent prognostic factors. (**B**) Lasso coefficient profiles of the 20 validated hub genes. (**C**) Forest plot of multivariate Cox regression model.

We evaluated the relationship between mRNA and PRAD prognosis, and 81 patients out of 499 were eliminated. Furthermore, 509 mRNAs were subjected to univariate Cox regression analysis. As a result, there were determined to be 111 biochemical recurrence-related mRNAs (*P* < 0.05). Simultaneously, 509 mRNAs were subjected to a log-rank analysis. There were determined to be 104 mRNAs associated with biochemical recurrence (*P* < 0.05). Next, we take the intersection of the results, and 72 genes were found. In addition, we excluded non-protein-coding genes from them and got 51 genes in the last. To punish every variable to screen variables, the Lasso Cox regression was used. Following that, the Lasso Cox regression was used to choose 20 mRNAs based on the minimal *λ* value (Figures 3A,B). After that, the My.stepwise R package was used to obtain the best candidate final model. Finally, seven TCGA-PRAD prognosis-related mRNAs that were significant were obtained: (*VWA5B2* + *ARC* +* SOX11* + *MGAM* + *FOXN4* + *PRAME* + *MMP26*) (Figure 3C). We next constructed a risk score formula based on the expression and Cox coefficient of the seven genes to predict the prognosis of PRAD:

$$\begin{array}{rl} \mathrm{Risk}\;\mathrm{Score}= & \;(0.21342\times \mathrm{VWA}5B2)+(-0.30113\times \mathrm{ARC})\\ & +(0.17896\times \mathrm{SOX}11)+(0.22222\times \mathrm{MGAM})\\ & +(0.18263\times \mathrm{FOXN}4)+(0.08836\times \mathrm{PRAME})\\ & +(-0.12725\times \mathrm{MMP}26) \end{array}$$

**Figure 4:**
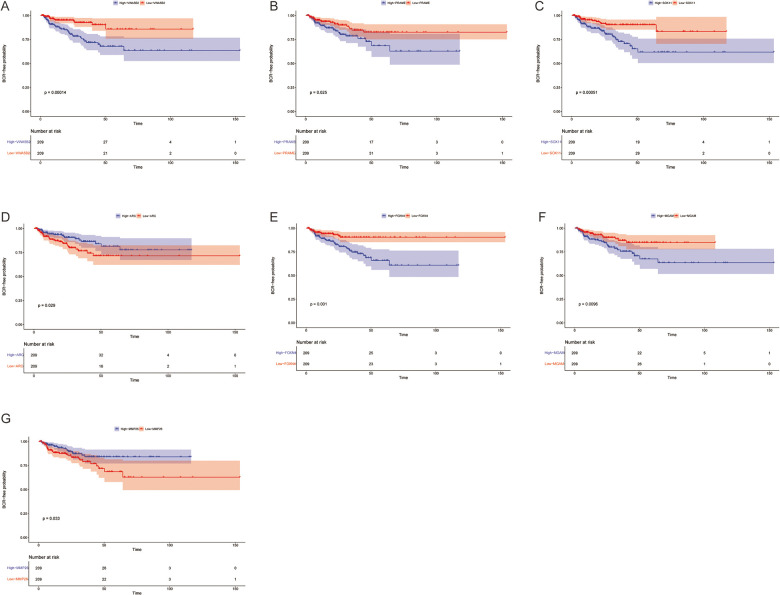
Biochemical recurrence free probability analysis of 7 genes. Gene changes of (**A**) *VWA5B2*, (**B**) *PRAME*, (**C**) *SOX11*, (**D**) *ARC*, (**E**) *FOXN4*, (**F**) *MGAM*, and (**G**) *MMP26* were significantly correlated with the biochemical recurrence free probability of prostate cancer patients.

**Figure 5:**
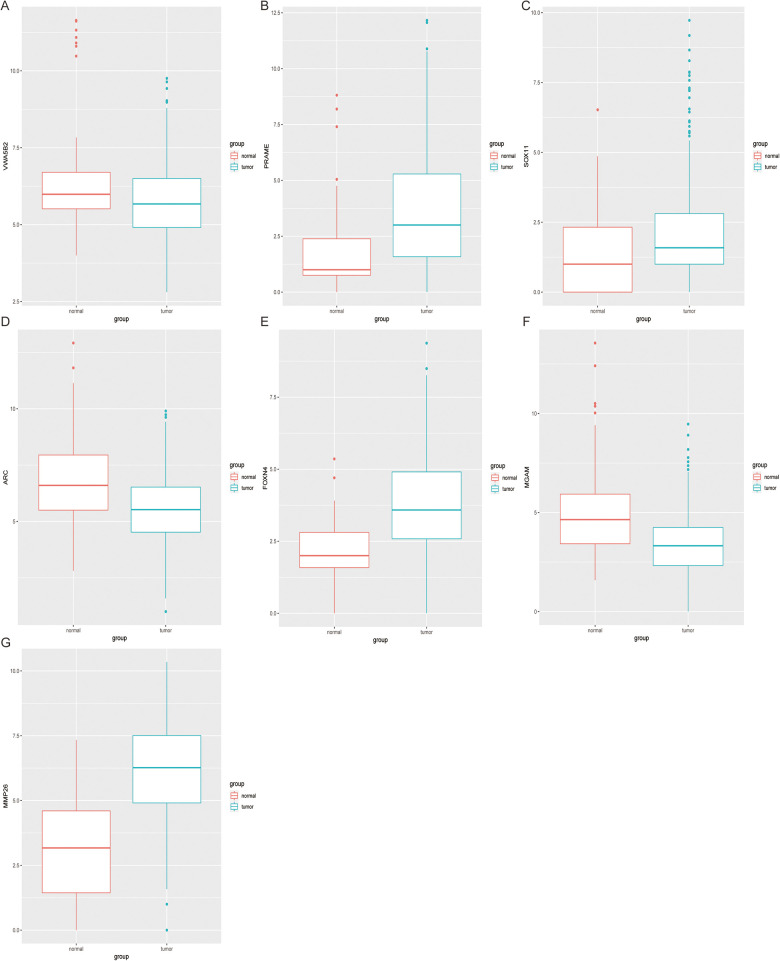
Expression difference analysis of seven genes. The boxplot shows the 7 genes (****A**–**G****) expression level between tumor tissues and adjacent non-tumor tissues.

**Figure 6:**
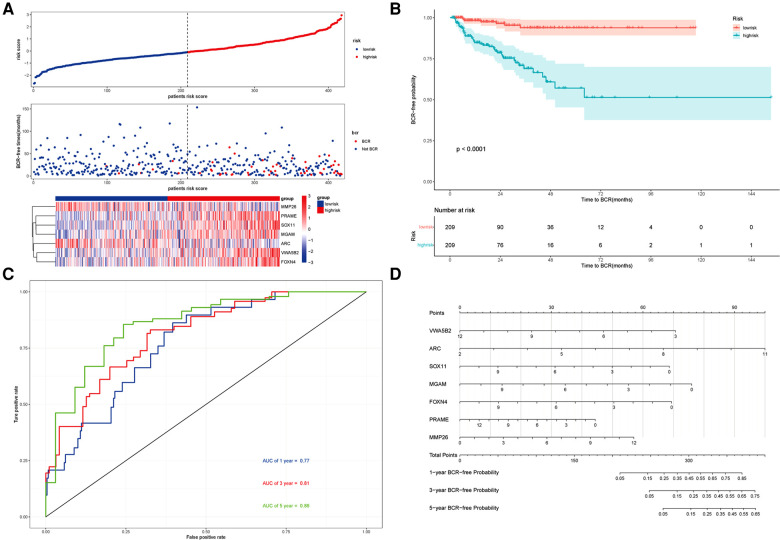
Prognosis analysis of the risk model and the nomogram. (**A**) Upper panel representing the distribution of risk scores for each, the middle panel showing the patient distribution with increasing risk value, and the lower panel representing the heatmap of expression of seven protein-coding genes. (**B**) Kaplan–Meier prognostic curve for the patients in PRAD between the high- and low-risk groups. (**C**) timeROC curve used to evaluate the accuracy of the seven protein-coding genes model. The 1-year, 3-year, and 5-year biochemical recurrence-free survival was 77%, 81%, and 86%, respectively. (**D**) Nomogram to predict the biochemical recurrence-free probability of PRAD.

Using this formula, the log_2_ value was used to calculate the expression level of the gene. Using the algorithm, the risk scores for each patient were then determined. Further analysis of the Kaplan–Meier plot curve showed the expression and prognosis of seven genes (Figures 4A–G). Also, the boxplot of the expression level of seven genes is shown in Figures 5A–G. According to the median score, the patients were separated into high- and low-risk groups. The risk score distribution and gene expression results revealed that patients in the high-risk group had a greater probability of biochemical recurrence than that in the low-risk group (Figure 6A). The Kaplan–Meier plot curve demonstrated that low-risk patients had a lower likelihood of biochemical recurrence than high-risk patients (*P* < 0.01) (Figure 6B). The timeROC curve was used to evaluate the sensitivity and specificity of the seven protein-coding gene model. The 1-year, 3-year, and 5-year AUCs of BCR-free probability were 77%, 81%, and 86%, respectively (Figure 6C).

### Nomogram with seven protein-Coding gene model

According to the seven protein-coding gene risk model, we use the RMS R package to draw the nomogram for BCR-free survival of PRAD (Figure 6D). The explanation of the nomogram is as follows: “point” is the score corresponding to a single variable, and the straight-line length of each variable reflects the contribution of each variable to the biochemical recurrence of PRAD. The total point is the total score obtained by accumulating the “score” corresponding to each variable. According to the total score obtained by each patient, we can correspond to the probability of BCR-free survival in 1-year, 3-year, and 5-year. In the nomogram, the greater the value of the “total score” of the patient, the BCR-free probability is greater and the BCR risk is smaller.

### Evaluation and external validation of the prognostic model

**Figure 7:**
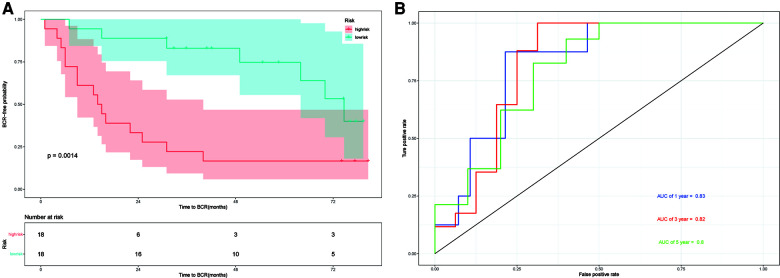
External validation in GSE46602. (**A**) Kaplan–Meier prognostic curve for the patients in GSE46602 between the high- and low-risk groups. (**B**) timeROC curve used to evaluate our model in GSE46602. The 1-year, 3-year, and 5-year BCR-free probability is 83%,82%, and 80%, respectively.

The GSE46602 dataset from the GEO database is used to evaluate our prognostic model. The Kaplan–Meier plot curve demonstrated that low-risk patients had a lower likelihood of biochemical recurrence than high-risk patients (*P* < 0.01) (Figure 7A). The timeROC curve was used to evaluate the sensitivity and specificity of the seven protein-coding genes model. The 1-year, 3-year, and 5-year AUCs of BCR-free probability were 83%, 82%, and 80%, respectively (Figure 7B). These results indicate that our prognostic model is valid.

### Verification of expression levels of seven genes between paired PRAD and adjacent nontumorous tissues

**Figure 8:**
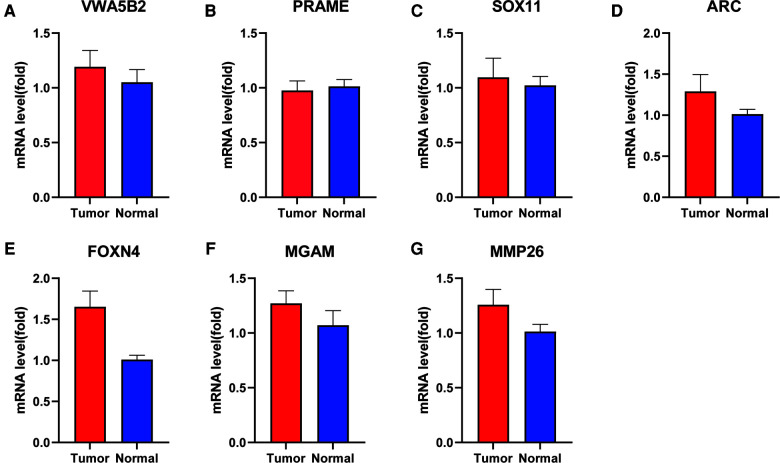
mRNA level of seven genes between para-tumor and PRAD tissues by qRT-PCR. (**A**) *VWA5B2*, (**B**) *PRAME*, (**C**) *SOX11*, (**D**) *ARC*, (**E**) *FOXN4*, (**F**) *MGAM*, and (**G**) *MMP26*.

To confirm the expression levels of the seven protein-coding genes, qRT-PCR was conducted in both paratumor and PRAD tissues. The results of qRT-PCR (Figure 8) revealed that expression levels of six genes tended to increase and that of one gene tended to decrease. The expression of *PRAME* is not obvious.

## Discussion

Prostate cancer is one of the three most prevalent urogenital system malignancies. BCR is related to tumor progression and metastasis in prostate cancer. However, the average survival time of patients with castration-resistant prostate cancer (CRPC) is only 16–18 months, with 90% developing distant metastasis (14). This better understanding of the relationship between BCR and eventual clinical progression is helpful to subsequently therapy for recurrent prostate cancer (15). Roehl et al. reported that BCR-free survival probability was substantially correlated with preoperative PSA, clinical tumor stage, Gleason total, pathological stage, and clinical therapy (16). According to a cohort Boorjian et al. conducted, positive margin was related to a higher incidence of BCR and local recurrence (17). The AUC was only 0.669% based on fusion biopsy in the standard D'Amico risk model (18). Kattan (19) established the first postoperative nomogram for biochemical recurrence after prostate cancer radical prostatectomy in 1999. Pretreatment PSA, Gleason grade, prostate capsular invasion, surgical margin status, seminal vesicle invasion, and lymph node status were included in their model. This model has been validated by a subsequent cohort, and the AUC is 0.772. However, this model is complicated; it is limited in clinical application. Cooperberg et al. created the UCSF cancer of the prostate risk assessment (CAPRA) score (20). Preoperative PSA, Gleason score, clinical T-stage, biopsy results, and age were included in their model. The concordance index of this model was 0.66. Our preliminary research has facilitated the development of a clinical model for predicting biochemical recurrence. Our preliminary model includes seminal vesicle invasion on MRI, the greatest MRI lesion's enormous diameter, and the ISUP grade of targeted fusion biopsy. The risk classification model's AUC is 0.72 (21). Lv et al. (22) proposed a nine-ferroptosis-related gene model. External validation was done using the TCGA and MSKCC cohorts. The 1-year, 3-year, and 5-year AUCs in the TCGA cohort were 0.680, 0.738, and 0.767, respectively. The 1-year, 3-year, and 5-year AUCs in the MSKCC group were 0.766, 0.729, and 0.726, respectively. Zhao et al. (23) proposed a three metabolic gene model. However, the 3-year and 5-year AUCs of this model were 0.739 and 0.72. We investigated the prognosis and function of key protein-coding genes in TCGA-PRAD in a systematic way. Based on the TCGA-PRAD dataset, 51 protein-coding genes were discovered in patients in TCGA-PRAD. Subsequently, a seven protein-coding gene prognostic model was identified and built. *FOXN4*, *MGAM*, *MMP26*, *ARC*, *SOX11*, *PRAME*, and *VWA5B2* were included in the seven protein-coding gene model.

According to the functional enrichment, hormone metabolism and cholesterol metabolism pathways have a significant role. Androgen hormones drive prostate cancer cell proliferation and progression by activating the androgen receptor. So, androgen deprivation therapy is the standard method for the treatment of prostate cancer. Together, the PI3K-AKT-mTOR pathway and androgen receptor can promote prostate cancer growth and treatment resistance (24). Also, these molecules connect lipid and cholesterol metabolism. Abnormal lipid metabolism and aberrant cholesterol synthesis are involved in the pathogenesis of prostate cancer. Ruffell et al. (25) proposed that macrophage reduction decreased androgen levels inside prostate cancers and restricted androgen receptor nuclear localization. Macrophages were cholesterol-rich and capable of transferring cholesterol to tumor cells. AR nuclear translocation was reduced by activation of Liver X Receptor (LXR)-β, which is the main factor of cholesterol metabolism. The mechanism of lipid metabolism may be a possible therapeutic strategy for prostate cancer. Zou et al. reported that Foxn4 regulates retinal progenitor fate and proliferation. The expression of Foxn4 is associated with the regulation of the development of tissues and organs (26). Pradeep et al. reported that MGAM could be a significant gene that drives oral squamous cell carcinoma (OSCC) development (27). MMP-26 and TIMP-4 may play an important role in the transformation of high-grade prostatic intraepithelial neoplasia (HGPIN) to invasive carcinoma and may potentially act as diagnostic indicators for early prostate cancer (28). In our model, MMP26 is the protective factor for the biochemical recurrence of prostate cancer. It needs further study and discussion. Bramham et al. reported that activity-regulated cytoskeletal-associated protein (Arc) is associated with synaptic plasticity (29). Neuroendocrine prostate cancer (NEPC) is highly aggressive and may emerge from prostate adenocarcinoma due to lineage plasticity, which is the end stage of prostate cancer. There are relatively few treatment choices, and the median overall survival is <1 year (30). Since then, we believe that ARC may play a role in the development of adenocarcinoma to NEPC. Several malignancies, including ovarian cancer and breast cancer, have been correlated to the aberrant upregulation of sox11. Howard et al. proposed that sox11 can stimulate SLUG expression in endocrine-resistant breast cancer by binding to its promoter, leading to the stimulation of epithelial-mesenchymal transition (EMT) and repression of ESR1 expression (31). PRAME is not found in normal tissues but is substantially expressed in numerous malignancies. In breast cancer, its high expression is associated with poor survival and is used as a prognostic marker (32). As for VWA5B2, there is no report at present. In summary, we hypothesize that these genes may play a role in various pathways in prostate cancer and require further study.

At present, few protein-coding gene prognostic models have been developed to predict the biochemical recurrence of prostate cancer. In comparison with the previous models, we have exploited more statistical methods to obtain prognosis-related genes in our model. Furthermore, the expression levels of seven genes were examined in PRAD tissues, which should be confirmed in the studies with larger sample sizes. In conclusion, we provide a novel protein-coding gene prognostic model for prostate cancer, which contributes to the prognostic evaluation of prostate cancer. Our model is more sensitive and accurate than the previous models, which facilitates its clinical application. At the same time, We will improve our model even more. For further research, we will explore possible pathways that contribute to the progression of prostate cancer.

## Conclusion

In this study, the differentially expressed genes in TCGA-PRAD were analyzed. The functional enrichment results imply that biochemical recurrence following radical prostatectomy was driven by underlying processes. A prognostic model composed of seven protein-coding genes (*FOXN4*, *MGAM*, *MMP26*, *ARC*, *SOX11*, *PRAME*, and *VWA5B2*) was established, and it was strongly associated with biochemical recurrence following radical prostatectomy in prostate cancer. The innovative protein-coding gene prognostic model may provide a new perspective for assessing the BCR of prostate cancer.

## Data Availability

The datasets presented in this study can be found in online repositories. The names of the repository/repositories and accession number(s) can be found in the article/Supplementary Material.
