# Supplementary material for: A seven-gene prognosis model to predict biochemical recurrence for prostate cancer based on the TCGA database
Source: Front Surg. 2022 Sep 5;9:923473. doi: 10.3389/fsurg.2022.923473 (PMC10226533; doi:10.3389/fsurg.2022.923473)
Supplement: Supplementary file 1 [file Table1.docx]

Supplemental TABLE 1 GO analysis of differential genes.

| Ontology | ID | Description |
| --- | --- | --- |
| CC | GO：0042627 | Chylomicron |
| CC | GO：0072562 | Blood microparticle |
| CC | GO：0034358 | Plasma lipoprotein particle |
| CC | GO：1990777 | Lipoprotein particle |
| CC | GO：0016324 | Apical plasma membrane |
| CC | GO：0034364 | High-density lipoprotein particle |
| CC | GO：0062023 | Collagen-containing extracellular matrix |
| CC | GO：0032994 | Protein-lipid complex |
| CC | GO：0045177 | Apical part of cell |
| CC | GO：0034361 | Very-low-density lipoprotein particle |
| MF | GO：0004866 | Endopeptidase inhibitor activity |
| MF | GO：0030414 | Peptidase inhibitor activity |
| MF | GO：0061135 | Endopeptidase regulator activity |
| MF | GO：0061134 | Peptidase regulator activity |
| MF | GO：0004867 | Serine-type endopeptidase inhibitor activity |
| MF | GO：0046873 | Metal ion transmembrane transporter activity |
| MF | GO：0004857 | Enzyme inhibitor activity |
| MF | GO：0015079 | Potassium ion transmembrane transporter activity |
| MF | GO：0033293 | Monocarboxylic acid binding |
| MF | GO：0005244 | Voltage-gated ion channel activity |
| BP | GO：0007389 | Pattern specification process |
| BP | GO：0003002 | regionalization |
| BP | GO：0042445 | Hormone metabolic process |
| BP | GO：0048562 | Embryonic organ morphogenesis |
| BP | GO：0042403 | Thyroid hormone metabolic process |
| BP | GO：0009952 | Anterior/posterior pattern specification |
| BP | GO：0023061 | Signal release |
| BP | GO：0048706 | Embryonic skeletal system development |
| BP | GO：0006590 | Thyroid hormone generation |
| BP | GO：0010951 | Negative regulation of endopeptidase activity |
