# Supplementary material for: A seven-gene prognosis model to predict biochemical recurrence for prostate cancer based on the TCGA database
Source: Front Surg. 2022 Sep 5;9:923473. doi: 10.3389/fsurg.2022.923473 (PMC10226533; doi:10.3389/fsurg.2022.923473)
Supplement: Supplementary file 2 [file Table2.docx]

Supplemental TABLE 2 KEGG analysis of differential genes.

| KEGG | Ascorbate bile aldarate biosynthesis | Retinal metabolism |
| --- | --- | --- |
|  |  | Steroid hormone biosynthesis |
|  |  | Ascorbate and aldarate metabolism |
|  |  | Pentose and glucuronate interconversions |
|  |  | Bile secretion |
| KEGG | Chemical drug carcinogenesis cytochrome | Metabolism of xenobiotics by cytochrome P450 |
|  |  | Chemical carcinogenesis-DNA adducts |
|  |  | Drug metabolism-cytochrome P450 |
|  |  | Drug metabolism-other enzyme |
|  |  | Chemical carcinogenesis-receptor activation |
| KEGG | Cholesterol ligand-receptor diabetes interaction | Neuroactive ligand-receptor interaction |
|  |  | Maturity onset diabetes of the young |
|  |  | Cholesterol metabolism |
| KEGG | Arachidonic serotonergic synapse acid | Serotonergic synapse |
|  |  | Arachidonic acid metabolism |
| KEGG | Collecting gastric pancreatic duct | Gastric acid secretion |
|  |  | Pancreatic secretion |
|  |  | Salivary secretion |
|  |  | Thyroid hormone synthesis |
|  |  | Collecting duct acid secretion |
