# Supplementary material for: A seven-gene prognosis model to predict biochemical recurrence for prostate cancer based on the TCGA database
Source: Front Surg. 2022 Sep 5;9:923473. doi: 10.3389/fsurg.2022.923473 (PMC10226533; doi:10.3389/fsurg.2022.923473)
Supplement: Supplementary file 3 [file Table3.docx]

Supplemental TABLE 3 Multivariate Cox regression analysis results

| Gene name | Coef | | Exp(coef) | | Lower.95 | Upper.95 | P value |
| --- | --- | --- | --- | --- | --- | --- | --- |
| VWA5B2 | 0.21342 | | 1.2379 | | 1.0114 | 1.5152 | 0.03849 |
| ARC | -0.30113 | | 0.7400 | | 0.6016 | 0.9102 | 0.00437 |
| SOX11 | 0.17896 | | 1.1960 | | 1.0447 | 1.3691 | 0.00949 |
| MGAM | 0.22222 | | 1.2489 | | 1.0405 | 1.4989 | 0.01702 |
| FOXN4 | 0.18263 | | 1.2004 | | 1.0409 | 1.3843 | 0.01205 |
| PRAME | 0.08836 | | 1.0924 | | 1.0006 | 1.1925 | 0.04839 |
| MMP26 | -0.12725 | | 0.8805 | | 0.7630 | 1.0161 | 0.08167 |
| Concordance=0.764(se=0.03) | | | | | | | |
| Likelihood ratio test | =54.74, | on7df | | p=0.000000002 | | | |
| Wald test | =52.55, | on7df, | | p=0.000000005 | | | |
| Score(logrank)test | =58.94, | on7df, | | p=0.0000000002 | | | |
