# Supplementary material for: A seven-gene prognosis model to predict biochemical recurrence for prostate cancer based on the TCGA database
Source: Front Surg. 2022 Sep 5;9:923473. doi: 10.3389/fsurg.2022.923473 (PMC10226533; doi:10.3389/fsurg.2022.923473)
Supplement: Supplementary file 4 [file Table4.docx]

Supplemental TABLE 4 Primers of seven genes

| VWA5B2-F | ACTCGGAGCAGACAGGTACT |
| --- | --- |
| VWA5B2-R | CCACCTTGAATGGAGCTGGT |
| PRAME-F | GTGGCAACAAGTGACTGAGACCTAG |
| PRAME-R | ATGCTGATGTATCGGCTCTGAATGG |
| SOX11-F | GTAGCAAATGGTGGCAAAGCAGAC |
| SOX11-R | TGGCGATGCGAGTTTCTATCAGTTC |
| ARC-F | TTCATCGTTCTGCCTTGTCCACTG |
| ARC-R | CCTGTGCCAGCCTTGAGGATTG |
| FOXN4-F | CTCAGCAGCCCCTCTCCTTCTC |
| FOXN4-R | GCTTGTCCACCTTCCTCCATTCTTC |
| MGAM-F | GAACATACCCAGCGTGCCATCC |
| MGAM-R | TGTCATTGAAGGTGAAGCCAAGGAG |
| MMP26-F | TCGGAATGGGACAGACCTACTTGAC |
| MMP26-R | TTCCTGGCGAGATGGAGGTGTC |
